# Creating topological exceptional point by on-chip all-dielectric metasurface

**DOI:** 10.1038/s41377-025-01955-2

**Published:** 2025-08-05

**Authors:** Cheng Yi, Zejing Wang, Yangyang Shi, Shuai Wan, Jiao Tang, Wanlin Hu, Zile Li, Yongquan Zeng, Qinghua Song, Zhongyang Li

**Affiliations:** 1 Electronic Information School, Wuhan University, Wuhan, 430072 China; 2 Tsinghua Shenzhen International Graduate School, Tsinghua University, Shenzhen, 518055 China; 3 Wuhan Institute of Quantum Technology, Wuhan, 430206 China

**Keywords:** Metamaterials, Sub-wavelength optics

## Abstract

Classified as a non-Hermitian system, topological metasurface is one of the ideal platforms for exploring a striking property, that is, the exceptional point (EP). Recently, creating and encircling EP in metasurfaces has triggered various progressive functionalities, including polarization control and optical holographic encoding. However, existing topological metasurfaces mostly rely on plasmonic materials, which introduce inevitable ohmic losses and limit their compatibility with mainstream all-dielectric meta-devices. Additionally, conventional free-space configurations also hinder the integration of multiple meta-devices in compact platforms. Here, an on-chip topological metasurface is experimentally demonstrated to create and engineer the topological phase encircling the EP in all-dielectric architecture. By massively screening the Si meta-atom geometry on the Si_3_N_4_ waveguide, a 2π-topological phase shift is obtained by encircling the EP. Through combining with the Pancharatnam-Berry (PB) phase, we decouple the orthogonal circular polarization channels and unfold the independent encoding freedom for different holographic generations. As a proof of concept, the proposed on-chip topological metasurface enables floating holographic visualizations in real-world scenarios, functioning as practical augmented reality (AR) functionalities. Such the all-dielectric on-chip scheme eliminates ohmic losses and enables compatible integration with other on-chip meta-devices, thus suggesting promising applications in next-generation AR devices, multiplexing information storage, and advanced optical displays.

## Introduction

Metasurface, as a type of 2D artificial material, has demonstrated significant potential in nanophotonic fields due to its exceptional capability to modulate the amplitude, phase, and polarization of light waves at the subwavelength scale^1–5^. Such optical field manipulation capability facilitates the development of various practical functions, including beam-steering^6–8^ and spectrum engineering^9–12^. In particular, its remarkable potential in optical encoding and modulation opens new avenues for the advancement of holographic meta-optics^13–17^. From a fundamental perspective, a metasurface device can serve as an open system, constantly exchanging and/or absorbing energy between the incident field and the resonant nanostructures. Such systems, classified as typical non-Hermitian systems, could exhibit a striking property, that is, the occurrence of a distinctive singular point, known as an exceptional point (EP)^18–20^. Specifically, located at the EP, the eigenvalues and eigenvectors would coalesce, thus triggering a sharp phase transition and leading to extraordinary optical responses. Based on this, metasurface, for its exceptional ability to manipulate light fields, is considered an ideal platform for creating and encircling an EP to obtain additional optical phase encoding degree of freedom (DoF)^21–23^. For instance, any arbitrary encircling path around the EP can provide a unique phase shift from 0 to 2π, known as topological phase. Hence, previous endeavors based on topological metasurfaces to engineer EP encircling have enabled various progressive functionalities^24,25^, including polarization control^26–29^, coherent perfect absorption^30–32^, and encoding holographic optical field^33,34^, etc.

However, state-of-the-art topological metasurfaces typically rely on plasmonic architectures to create an EP, which inherently introduces considerable ohmic losses due to the absorption properties of metals. It severely diminishes the overall optical efficiency and restricts it from being compatible with the mainstream meta-devices of all-dielectric counterparts. Moreover, the typical free-space configurations also hinder the integration of multiple meta-devices in compact optical platforms.

Recently, the invention of metasurface integrated onto optical waveguides has emerged as a promising alternative for next-generation miniature optical devices^35–38^. Unlike traditional metasurface designed for free-space optics, on-chip metasurface allows linearly polarized light to be coupled into the waveguide via end-fire coupling, after which it propagates as guided light within the waveguide. This platform offers the ability to manipulate guided waves, bridging the conversion between free-space light and on-chip guided waves. Hence, creating an EP encircling in the all-dielectric environment and on-chip scheme is highly desirable to remarkably reduce intrinsic ohmic losses and improve its integration compatibility, but remains an unexplored frontier to the best of our knowledge.

Here, we propose and experimentally demonstrate an all-dielectric on-chip metasurface patterned on a waveguide, which engineers the topological phase by encircling the EP for meta-optics holography. Specifically, by massively screening the Si meta-atom shape on the Si_3_N_4_ waveguide and scanning its geometric parameters, we successfully create an EP in the all-dielectric environment and achieve a 2π-accumulated topological phase around the EP. By incorporating the acquired topological phase with the Pancharatnam-Berry (PB) phase, we fully decouple and unfold the independent encoding DoF for different circular polarized light (CPL). As a proof of concept, vectorial holographic images can be independently imparted to left-handed circular polarization (LCP) and right-handed circular polarization (RCP) channels and actively switched for human eyes as AR meta-display functionality. Such an all-dielectric on-chip propagation scheme effectively eliminates the intrinsic ohmic losses associated with metallic materials, while also being free from any interference of zero-order diffraction background in the projected AR meta-holograms, thereby enhancing the display clarity and quality. Overall, our demonstration is an original attempt to integrate non-Hermitian characteristics into an all-dielectric, on-chip metasurface platform, enabling precise topological phase control and significantly expanding the encoding degrees of freedom. The proposed all-dielectric on-chip topological metasurface further offers distinct advantages by enhancing encoding capacity through polarization decoupling, while maintaining excellent compatibility with integrated photonic platforms. We believe it can find promising paths toward next-generation wearable AR devices, multiplexing information storage, and advanced optical display technologies.

## Results

**Fig. 1:**
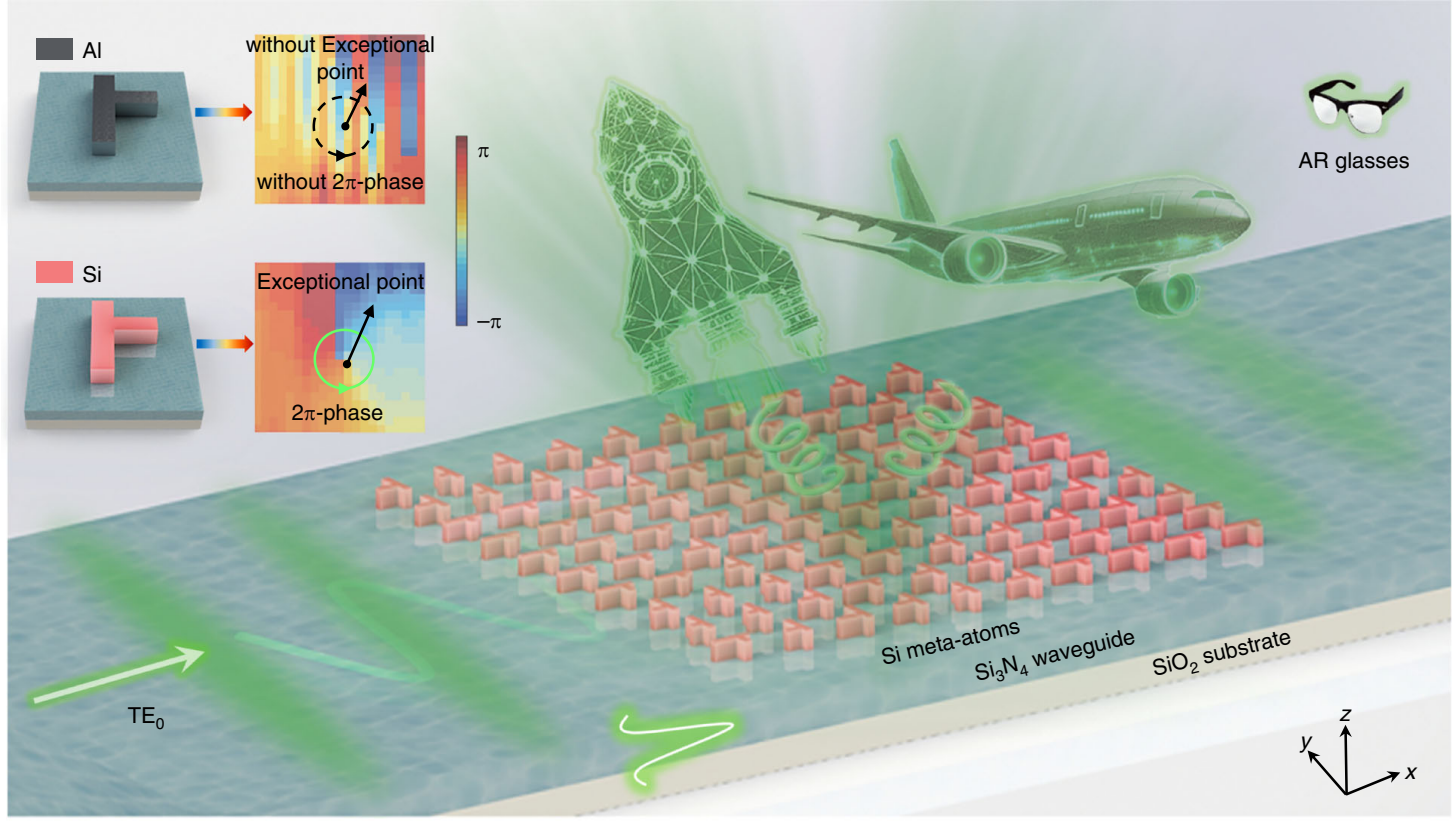
**Schematics of the on-chip all-dielectric metasurface based on topological phase encoding for dual-channel holographic display**. The on-chip metasurface is integrated onto the waveguide sitting on a transparent substrate to project LCP/RCP holographic images. The insets illustrate the phase distributions of the extracted LCP component for dielectric (Si) and metal (Al) materials

Figure 1 schematically illustrates the on-chip metasurface incorporating all-dielectric topological meta-atoms into the waveguide for switchable dual-channel vectorial holography. Particularly, the insets provide the representation of simulations for the on-chip designed Si structure, which exhibits topological characteristics, revealing an EP at a wavelength of 525 nm. When encircling the EP along an arbitrary path, it produces a 2π-phase accumulation, known as the topological phase. In contrast, if the counterpart structure is made of metallic material, for instance, aluminum (Al), no topological EP phenomenon is observed. These suggest that the discovered topological properties are inherent to the proposed all-dielectric structures, which cannot simply be duplicated in metallic counterparts (more details can be found in Supplementary Section S1, Supplementary Information). Moreover, by integrating the topological phase surrounding the EP with the PB phase, the on-chip metasurface achieves complete independent control over two circularly polarized channels. As a proof of concept, vectorial holographic images can be projected into the human eyes or smartphones for AR meta-display.

**Fig. 2:**
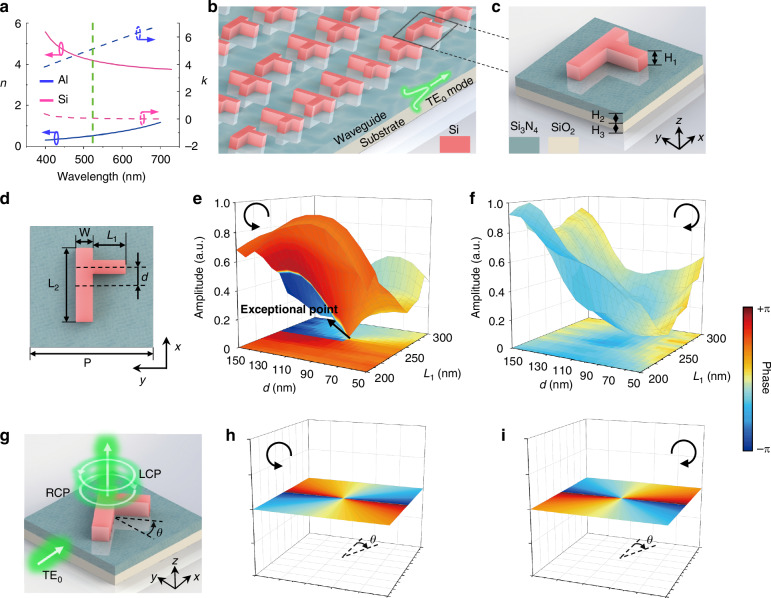
**Numerical analysis of the EP with on-chip all-dielectric topological metasurfaces**. **a** The complex refractive indices of Si and Al. The green dashed line marks the operating wavelength of 525 nm. Here, *n* and *k* denote the real and imaginary parts of the refractive index, respectively. **b** Schematic illustration of the on-chip all-dielectric metasurface with topological meta-atoms. **c** Perspective view of the Si meta-atom positioned on the top of a Si_3_N_4_ waveguide and SiO_2_ substrate. **d** Top view of the designed dielectric meta-atom. The dimensions are L_2_ = 440 nm, W = 70 nm, P = 500 nm. **e**, **f** Simulated extraction coefficients for LCP and RCP, covering the parameter space of *L*_1_ ∈ [200 nm, 300 nm] and *d* ∈ [50 nm, 150 nm]. An EP is obtained at (*L*_1_, *d*) = (250 nm, 90 nm) in LCP. **g** Perspective view of the meta-atom with a rotation angle *θ*. **h**, **i** Theoretical PB phase distributions for the on-chip metasurface with *θ* ∈ [0, 2π]

Notably, the proposed all-dielectric topological metasurface exhibits a notable advantage in eliminating the ohmic losses relative to metallic structures. As shown in Fig. 2a, the dielectric material of Si exhibits nearly lossless performance (*k* = ~0.05) at the broadband visible wavelengths, while the losses of metallic materials, such as Al (*k* = ~5), are remarkably higher. More detailed discussions regarding the complex refractive indices of Si and common metallic materials are provided in Supplementary Section S2, Supplementary Information. It is noteworthy that compared to the conventional ohmic-loss-induced non-Hermitian system, here the non-Hermitian characteristics of the on-chip dielectric metasurface arise from the fact that, the guided waves within the waveguide are only partially extracted and the rest of the optical energy would continue to propagate along the waveguide. To create a topological EP using an on-chip all-dielectric metasurface (Fig. 2b), we massively screened various structural shapes for meta-atom candidates (such as an F-shaped meta-atom in Supplementary Section S3, Supplementary Information). Eventually, considering both fabrication feasibility and the ease of manipulating EP, as shown in Fig. 2c, the T-shaped Si meta-atoms (H_1_ = 350 nm) are chosen and designed to be placed on the top of a high-refractive- index planar Si_3_N_4_ (*n* = ~2.05, H_2_ = 220 nm) waveguide with an underneath SiO_2_ substrate (H_3_ = 500 μm). Through fully studying the structural parameters of the meta-atoms, we found that *L*_1_ and *d* are two primary geometric variables for potentially creating the desired EP response, as shown in Fig. 2d. More detailed discussions on the influence of the refractive index of dielectric materials on EPs can be found in Supplementary Section S4, Supplementary Information. Here, *L*_1_ represents the length of the meta-atom arm and *d* denotes the displacement of the meta-atom arm from the center along the x-axis. Further details regarding the on-chip waveguide configuration can be found in Supplementary Section S5, Supplementary Information.

To theoretically demonstrate the existence of a topological EP, we conduct the numerical simulation by using the finite-difference time-domain (FDTD) method for the proposed all-dielectric on-chip metasurface. The incident light of TE_0_ mode propagates within the Si_3_N_4_ waveguide along the x-direction, which can be regarded as an in-plane y-linearly polarized light. Each meta-atom placed on the waveguide serves as an out-coupling antenna to extract the guided wave. Through exploring the parametric space via varying the parameters *L*_1_ and *d*, the corresponding extracted light exhibits distinct trends of phase and amplitude evolution for different circular polarizations. After elaborately scanning the two structural parameters, we eventually locate a topological EP occurring at (*L*_1_, *d*) = (250 nm, 90 nm) for 525 nm-wavelength light, as shown in Fig. 2e. To verify the degeneracy at the EP, we analyzed the azimuth and ellipticity angles of the light field within the parameter space, which shows that around the EP, the degenerate state exhibits circular polarization state to be consistent with the topological characteristics. More evidence and discussions are available in Supplementary Section S6, Supplementary Information.

On the one hand, for the LCP component, the extraction channel is completely suppressed at the EP, resulting in no optical extraction scenario (mimicking the perfect absorption phenomenon in the metallic counterpart). Accordingly, the LCP phase distribution in the parameter space defined by (*L*_1_, *d*) exhibits the classic topological protection to provide the topological phase, that is, when encircling the EP along any arbitrary closed path, the extracted phase maintains a full 2π-phase accumulation. On the other hand, for the RCP component extraction channel, a nearly flat distribution in the phase evolution is exhibited (Fig. 2f), thus indicating the EP absence for the RCP case. This distinction highlights the fundamental difference between non-Hermitian and Hermitian systems: the imparting phase mechanism in Hermitian systems exerts identical effects on both LCP and RCP components; in contrast, the topological phase from EP in the non-Hermitian system only impacts one particular polarization during the on-chip extraction process. Hence, the topological phase encoding allows for the possibility of fusion with the PB phase modulation. By exerting an angular rotation of *θ* to meta-atom (Fig. 2g), PB phase encoding would impart opposite phases to the extracted LCP and RCP from the waveguide. The theoretical LCP (Fig. 2h) and RCP (Fig. 2i) phase distributions exhibit the twofold relationship with the rotation angle *θ* and verify the opposite phase imparting in the on-chip scheme. Further details can be found in Section S7, Supplementary Information.

**Fig. 3:**
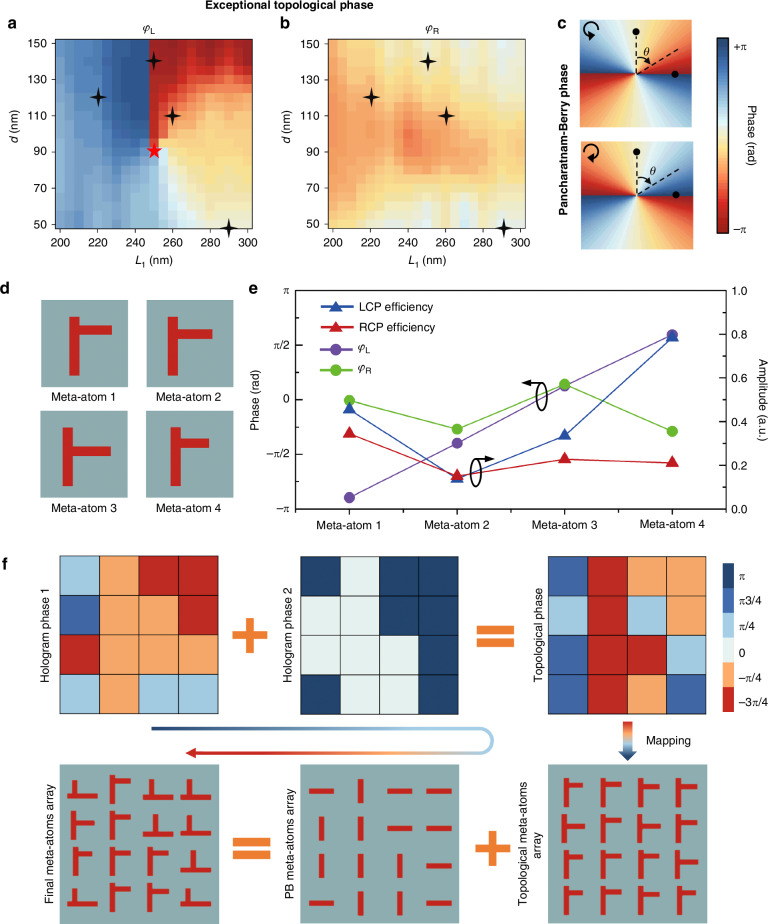
**Design of the on-chip topological metasurface for CPLs decoupling**. Simulated topological phase distributions for the extracted LCP component *φ*_L_ (**a**) and RCP component *φ*_R_ (**b**). **c** Theoretical distributions of the PB phase. **d** Schematic diagram of the four designed meta-atoms. **e** Simulated phases and extraction efficiencies of the four selected meta-atoms for different CPLs. **f** The design process of the on-chip topological metasurface array to generate predefined holographic phase profiles

Hence, by leveraging the fusion of the topological phase and PB phase, we propose a design principle for achieving the vectorial holography based on the on-chip topological metasurface operating around EP (Fig. 3). As clearly plotted and compared in Fig. 3a, b, the phase distribution of the extracted LCP component *φ*_L_ in parameter space (*L*_1_, *d*) shows typical phase shift trending surrounding the EP (denoted by a red star); in contrast, for the RCP component, the corresponding phase distribution *φ*_R_ is relatively flat, which suggests the possibility to fully decouple LCP and RCP encoding freedom by combining with PB phase (Fig. 3c). Additional discussion on the existence of the EP can be found in Supplementary Section S8, Supplementary Information.

For the holographic optical field encoding based on topological phases, four on-chip meta-atoms (Fig. 3d) with different structural parameters (denoted by black stars in Fig. 3a) are designed to exhibit a notable π/2 phase step. Figure 3e summarizes the simulated phase and extraction efficiency of the above four meta-atoms for LCP and RCP, respectively. It is observed that the phase distribution of *φ*_L_ (purple circles in Fig. 3e) exhibits a linear increment covering 2π span with π/2 interval, while the phase distribution of *φ*_R_ appears nearly uniform (green circles in Fig. 3e).

Next, we present the phase array design to encode two hologram phase maps for LCP and RCP into a single meta-atom array (Fig. 3f). Initially, we utilize a Gerchberg-Saxton (GS) algorithm to generate the corresponding phase distributions based on the target images of LCP and RCP channels. More detailed information about the GS algorithm can be found in Supplementary Section S9, Supplementary Information. For the LCP channel (Hologram phase 1), the designed four-step phase array is governed by both the topological phase and PB phase; in contrast, for the RCP channel (Hologram phase 2), the designed two-step phase array is solely determined by the PB phase. Therefore, the required topological phase can be derived by summing Hologram phases 1 and 2. By precisely tuning the calculated topological phase step to align with our selected meta-atoms, we obtain the corresponding topological meta-atoms distribution, which is responsible for imparting the topological phase. Eventually, with the introduction of different rotation angles to achieve the PB phase, the finalized meta-atoms array is obtained to decouple the LCP/RCP light and independently encode two vectorial holographic channels. Notably, compared with conventional methods for decoupling LCP and RCP channels^39,40^, our strategy enables the extension of phase modulation into a broadband response regime.

**Fig. 4:**
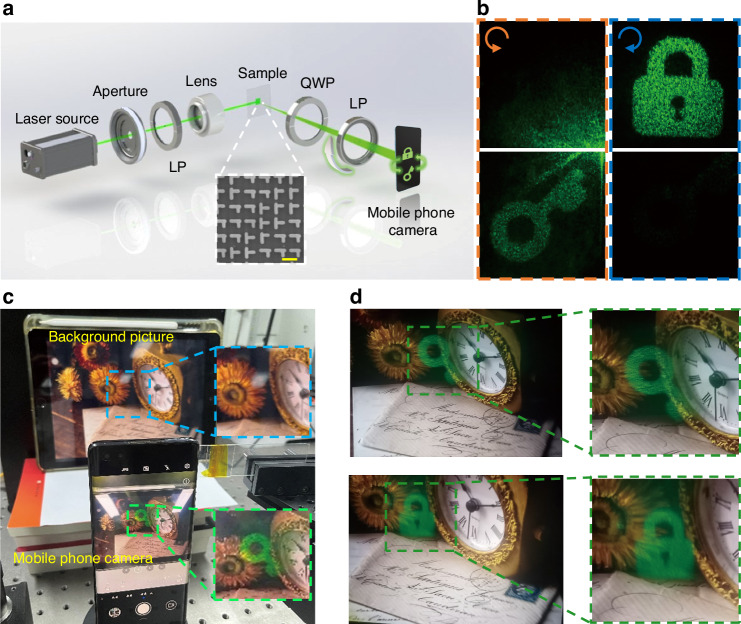
**Experimental characterization for the on-chip meta-holography and AR demonstration**. **a** Optical setup to characterize the on-chip meta-holography. LP: linear polarizer. QWP: quarter-wave plate. Inset: SEM image of the metasurface sample. Scale bar: 500 nm. **b** Measured LCP holographic image of “Key” and RCP holographic image of “Lock”. **c** Actual experimental setup for the AR demonstration. The mobile phone camera captures virtual images floating on the background (green dashed frame) with no images present in the background picture (blue dashed frame). **d** Experimentally captured AR images of a green “Key” and a green “Lock” floating on top of the background picture

As the experimental validation, the on-chip metasurface sample is fabricated by using plasma-enhanced chemical vapor deposition (PECVD), conventional electron-beam lithography (EBL) techniques, etc. The optical setup for AR holographic characterization is established, as depicted in Fig. 4a, with an inset scanning electron microscope (SEM) image of the metasurface sample. The incident laser is coupled into the waveguide simply in an end-fire configuration, and the projected holographic image can be directly captured by a mobile phone camera. By employing polarization analyzers to selectively transmit distinct CPLs, different holographic images can be switched accordingly. When the output channel is set to LCP, a target holographic image of “Key” appears, while switching to RCP results in a “Lock” holographic image (Fig. 4b). Such experimental holographic observation confirms that the designed all-dielectric on-chip topological metasurface effectively achieves decoupling of LCP and RCP, with high-contrast distinction and almost no cross-talk between the two channels. More details for fabrication processing and measurements can be found in Methods and Supplementary Section S10, Supplementary Information.

As a proof of concept, we further present its AR functionality in a real-world environment with its experimental setup in Fig. 4c. Notably, no holographic image appears in the background picture, however, the meta-hologram is projected and captured by the mobile phone camera, seemingly floating above the background. Specifically, as shown in Fig. 4d, the dual-channel AR meta-holograms (“Key” and “Lock”) are alternatively switched to present as a floating virtual image in the background provided by another display screen. Due to the high transparency of the all-dielectric architecture and the unique on-chip propagation scheme without zero-order diffraction interference, the projected AR meta-holograms remain clearly visible to the observer with satisfactory imaging clarity and contrast, despite of minor stray light noise. This suggests the significant potential of on-chip all-dielectric topological meta-devices in advanced AR meta-display technologies and applications.

## Discussion

In summary, we propose and experimentally demonstrate an on-chip topological metasurface integrated on a waveguide to create an EP in all-dielectric architecture, thus eliminating the ohmic losses inherent to metallic materials. Through comprehensively scanning the structural parameters of Si meta-atoms at the nanoscale, we create and encircle an EP without involving any metallic components to obtain a 2π topological phase, serving as an additional DoF in optical phase encoding. By incorporating the PB phase, we achieve full decoupling of orthogonal circular polarization channels, enabling switchable dual-channel meta-holography. Ultimately, we successfully enable holographic virtual images floating in the actual-world scene as AR meta-displays. Thanks to the all-dielectric and unique on-chip propagation scheme, the proposed meta-devices do not suffer from any zero-order diffraction interference to the observer and facilitate the potential integration of multiple on-chip components in compact platforms. Overall, this platform offers a promising path toward next-generation display and storage applications, including wearable AR displays, information storage, and optical multiplexing.

## Materials and methods

### Sample fabrication

A 220 nm-thick Si_3_N_4_ layer was deposited on a 500-µm-thick fused silica substrate using PECVD. Following this, a 350-nm-thick Si layer was deposited on the Si_3_N_4_ waveguide. To pattern the metasurface, polymethyl methacrylate (PMMA) resist was spin-coated onto the Si layer and baked at 150 °C for 3 min. A conductive polymer was then spin-coated to mitigate charge accumulation during subsequent processing. The sample was exposed by EBL (Raith eLINE Plus, 20 kV), followed by development in a solution for 80 s and rest in the IPA solution for 60 s. A 20-nm-thick chromium (Cr) layer was then deposited via thermal evaporation to serve as an etching mask. After the PMMA resist was removed using a lift-off process in acetone, the Cr mask pattern was transferred to the Si layer through reactive ion etching. Finally, the Cr layer was removed using a Cr etchant, leaving the final metasurface structure.

### Numerical simulation

Numerical simulations were carried out using the FDTD method to analyze the performance of the proposed structure. Periodically arranged Si meta-atoms were placed above the Si_3_N_4_ (*n* = ~2.05) waveguide on the SiO_2_ substrate (*n* = 1.45) to investigate the electric-field intensity distributions of the extracted light. The simulation was conducted in three dimensions, applying perfectly matched layer boundary conditions in the x-direction and z-direction and periodic boundary conditions along the y-direction. The propagating guided wave in the waveguide is the transverse electric mode (TE_0_).

### Optical measurement

The on-chip holographic images were captured using the optical setup shown in Fig. 4a. The laser was passed through an aperture diaphragm to control the spot size, followed by a linear polarizer and a set of lenses to couple the light into the waveguide from the sample edge. To obtain the holographic images with different circular polarization states, a combination of the QWP and LP was placed between the mobile phone camera and the metasurface. The holographic images shown in Fig. 4b were reasonably processed with brightness/contrast enhancement. For the AR meta-holography (Fig. 4d), the AR holographic images floating on top of the background in the real-world environment can be directly captured by utilizing the mobile phone camera.

## Supplementary information

Supplementary Information for Creating Topological Exceptional Point by On-Chip All-Dielectric Metasurface

## Data Availability

Data that support the findings of this study are available from the corresponding authors upon reasonable request.
