# Supplementary material for: Creating topological exceptional point by on-chip all-dielectric metasurface
Source: Light Sci Appl. 2025 Aug 5;14:262. doi: 10.1038/s41377-025-01955-2 (PMC12325960; doi:10.1038/s41377-025-01955-2)
Supplement: Supplementary file 1 — Supplementary Information for Creating Topological Exceptional Point by On-Chip All-Dielectric Metasurface [file 41377_2025_1955_MOESM1_ESM.pdf]

Supplementary Information for

Creating Topological Exceptional Point by On-Chip All-Dielectric Metasurface

*Cheng Yi[1, †], Zejing Wang[1, †], Yangyang Shi[1], Shuai Wan[1], Jiao Tang[1], Wanlin Hu[1], Zile Li[1], Yongquan Zeng[1], Qinghua Song[2, *], Zhongyang Li[1, 3, *]*

[1] *Electronic Information School, Wuhan University, Wuhan 430072, China*
[2] *Tsinghua Shenzhen International Graduate School, Tsinghua University, Shenzhen, 518055, China*
[3] *Wuhan Institute of Quantum Technology, Wuhan 430206, China*
[†] *These authors contributed equally to this work.*
* *Email: Z.Y.L. zhongyangli@whu.edu.cn; Q.S. song.qinghua@sz.tsinghua.edu.cn*

This PDF file includes:

Supplementary Text
Figs. S1 to S13

S1. Scanning results for Al meta-atoms on a Si_3N_4 waveguide

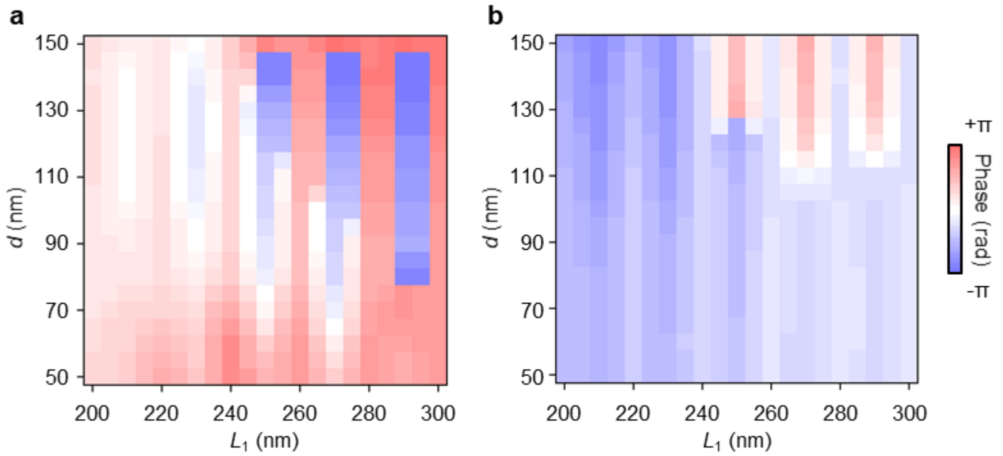

Fig. S1. The phase distributions of the extracted LCP (**a**) and RCP (**b**) components from the guided waves.

Under the same simulation conditions, when the material of the meta-atoms integrated onto the waveguide is replaced with Al, the phase distribution of the extracted LCP component no longer exhibits topological characteristics (Fig. S1a), and the RCP component similarly lacks topological features (Fig. S1b). This implies that, the topological properties of the selected design are intrinsic to the dielectric environment and cannot be readily transferred to metallic structures at the same operating condition.

S2. Comparison of the complex refractive index between silicon and common metallic materials.

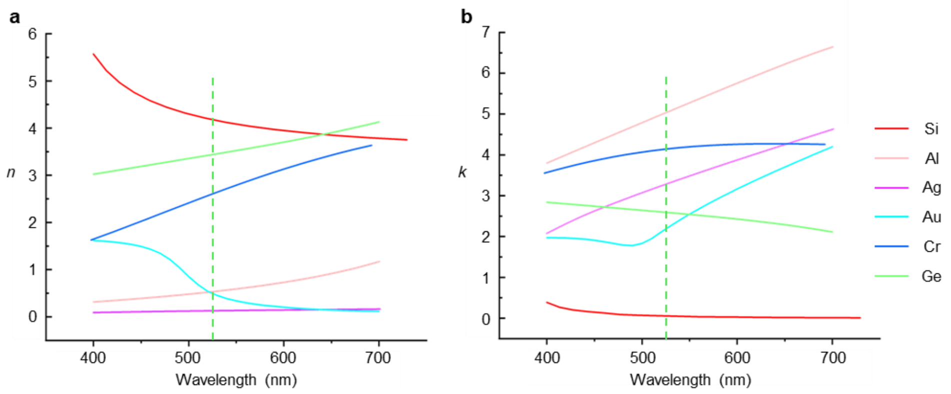

Fig. S2. Real (**a**) and imaginary (**b**) parts of the complex refractive indices of silicon and several common metallic materials in the visible spectrum.

As shown in Fig. S2, in addition to the initial comparison between silicon (Si) and aluminum (Al), we now provide a broader survey of the complex refractive indices (both real and imaginary parts) for representative metals frequently employed in metasurface designs, including argentum (Ag), aurum (Au), chromium (Cr), and germanium (Ge).

At the operating wavelength used in our work (indicated by the green dashed line), the imaginary part (k) of Si's refractive index is significantly lower than that of metallic materials. Notably, even Au, which exhibits the lowest imaginary refractive index among the surveyed metals, still possesses an imaginary refractive index nearly 40 times higher than that of silicon. This spectral comparison across the visible range clearly confirms that Si, as a dielectric material, offers consistently lower absorption

loss compared to metallic counterparts.

S3. Numerical simulation results of the EP of the F-shaped structure

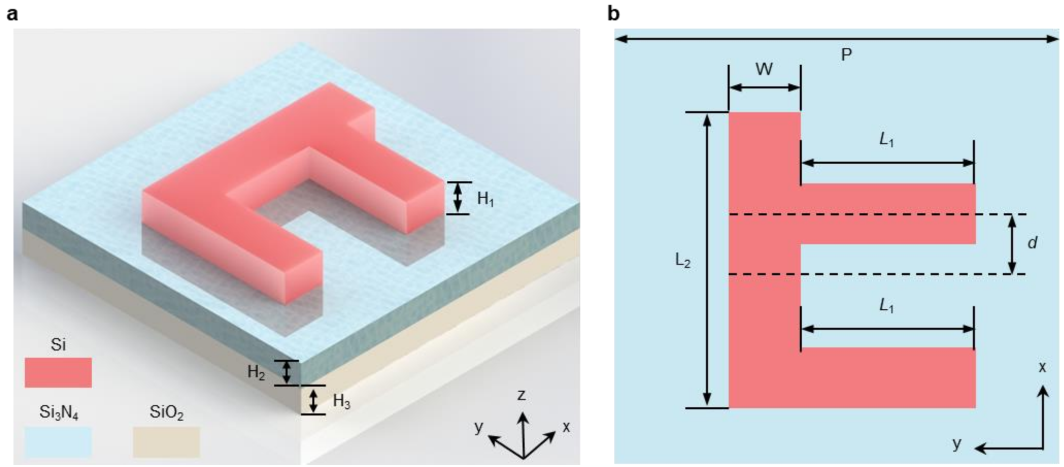

Fig. S3. **a**, Perspective view of the Si meta-atom positioned on the top of a Si_3N_4 waveguide and SiO_2 substrate. **b**, Top view of the designed dielectric meta-atom.

In principle, any geometry that facilitates the extraction of guided waves and allows for the construction of the relevant parameter space within a non-Hermitian framework is compatible with our on-chip topological metasurface platform. For instance, alternative configurations such as the F-shaped structure (Fig. S3) can also be explored within the same conceptual framework and exhibit the EP behavior within the designed parameter space. More complex geometries may further expand the design flexibility and functional diversity of the proposed approach.

To validate our hypothesis, as illustrated in Fig. S3a, we designed F-shaped Si meta-atoms (H_1 = 350 nm) placed atop a high-refractive-index planar Si_3N_4 waveguide layer ($n \approx 2.05$, H_2 = 220 nm), supported by a SiO_2 substrate (H_3 = 500 μm). As shown in Fig. S3b, two primary geometric parameters, L_1 and d, are selected to explore the

emergence of the desired EP response. Here, L_1 represents the length of the meta-atom arm, while d denotes the lateral displacement of the arm from the center along the x-axis. The other structural parameters were fixed as L_2 = 440 nm, W = 70 nm, and P = 500 nm.

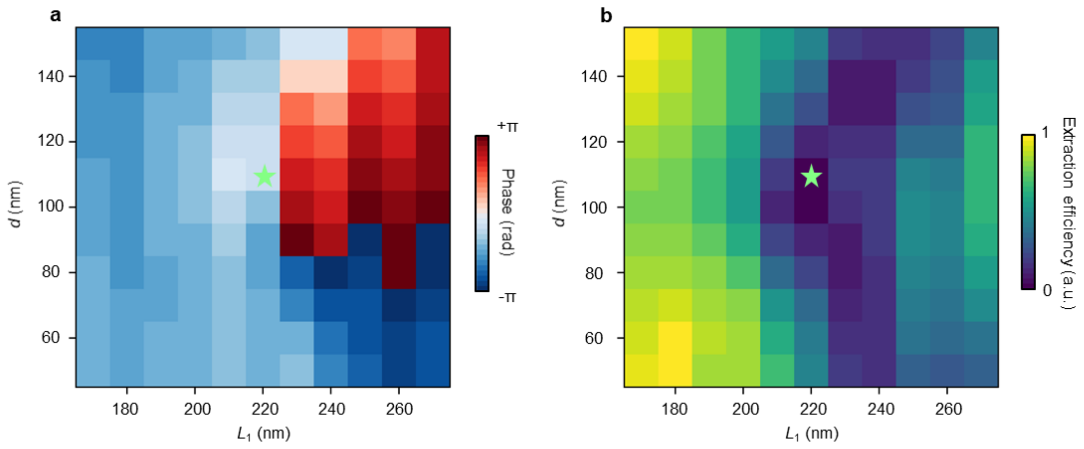

Fig. S4. Simulated topological phase (**a**) and extraction efficiency (**b**) distribution for the extracted LCP component from the F-shaped structure at 515 nm.

As shown in Fig. S4, we successfully identified an EP under the configuration of F-shaped meta-atoms in parameter space (L_1, d), highlighting the generality of our proposed design strategy.

S4. Influence of the refractive index of dielectric materials on EPs

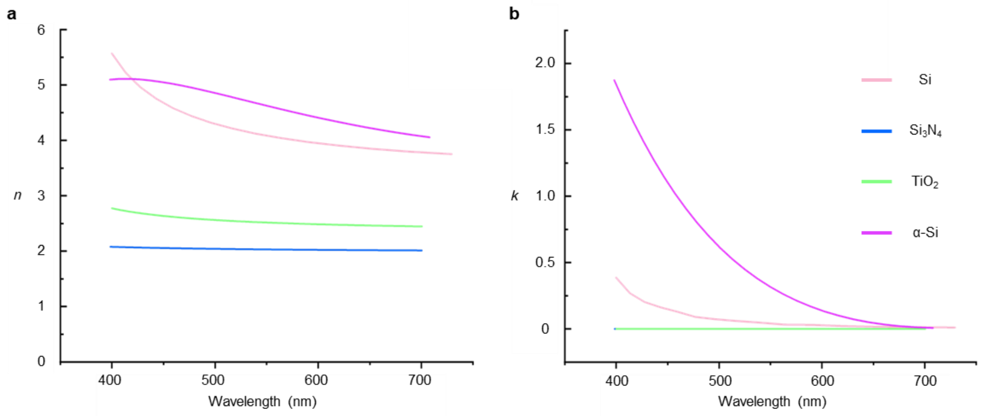

Fig. S5. Real (**a**) and imaginary (**b**) complex refractive indices of common dielectric materials in the visible spectrum.

As shown in Fig. S5b, the α-Si extinction coefficient in the visible region is actually higher than that of Si at the operating visible wavelength of 525 nm. Although α-Si becomes more transparent at longer wavelengths (with k approaching zero), its absorption at 525 nm is significantly greater than that of Si, making Si a more appropriate choice in terms of balancing high refractive index and low absorption loss at our working wavelength.

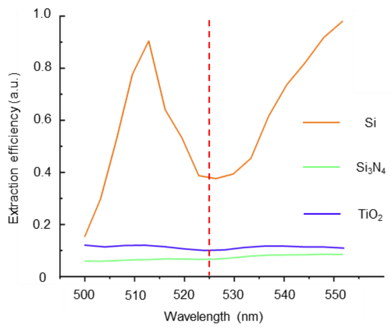

Fig. S6. Guided waves extraction efficiency of different dielectric materials under identical structural

configurations.

Additionally, although other dielectric materials such as titanium dioxide (TiO_2) and Si_3N_4 exhibit nearly zero extinction coefficients (the blue curve representing Si_3N_4 overlaps with the green curve representing TiO_2 in above Fig. S5), their performance in terms of guided waves extraction efficiency is significantly lower (see Fig. S6) when used in the same metasurface structure due to its lower refractive index. This reduced extraction efficiency limits their effectiveness for light manipulation in our on-chip system and would hinder both the demonstration and proof of concept of the holographic functionality we aim to achieve.

In summary, Si is selected as a more favorable material at the operational wavelength when compared to α-Si and Si_3N_4, due to its lower extinction coefficient relative to α-Si and superior extraction efficiency compared to Si_3N_4.

To investigate the wavelength dependence of EPs in different materials, we performed a comparative analysis of the identified EPs in both Si and Si_3N_4 configurations. As shown in Fig. S7, we analyzed the phase distributions of the extracted LCP component for both Si and Si_3N_4-based structures at wavelengths of 525 nm and 545 nm, corresponding to the respective EP conditions observed for each material system. Specifically, at 525 nm (Fig. S7c), the Si-based configuration exhibits a clear EP, while no EP is observed in the Si_3N_4-based configuration (Fig. S7a). Conversely, at 545 nm,

an EP appears in the Si_3N_4-based structure (Fig. S7b), but not in the Si-based counterpart (Fig. S7d). These results demonstrate that the location of the EP is sensitive to the material's refractive index, leading to a shift in the corresponding wavelength. However, the EP itself remains robust and does not disappear, thereby confirming the persistence of non-Hermitian behavior across different dielectric material platforms. This further underscores the generality and adaptability of our proposed on-chip topological metasurface design.

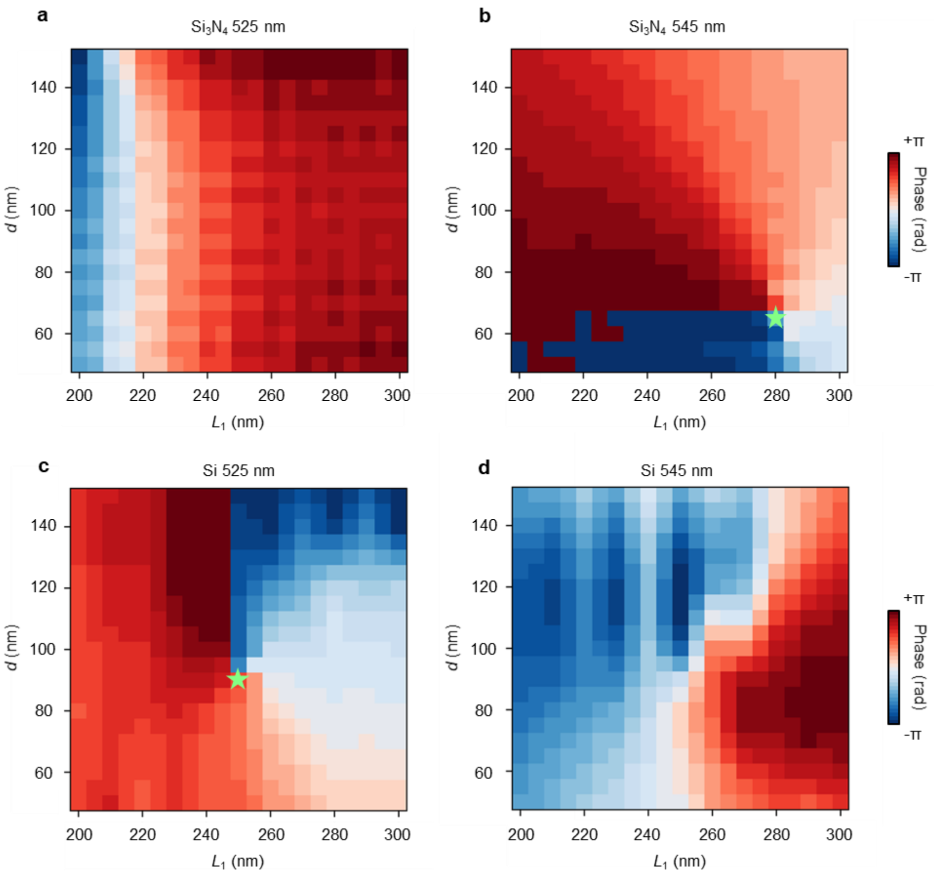

Fig. S7. Phase distributions of the LCP component for Si_3N_4 under 525 nm (**a**) and 545 nm (**b**) vs. Si under 525 nm (**c**) and 545 nm (**d**).

S5. Characteristics of on-chip waveguide configuration

First, one of the primary factors to consider is the method of light excitation. In our proposed on-chip metasurface platform, excitation is achieved via end-fire coupling of linearly polarized light into the waveguide. In contrast to free-space metasurfaces, which require careful consideration of varying incident angles and polarization states, our in-plane waveguide configuration constrains the excitation to a fixed fundamental guided mode. This results in more stable and controllable input conditions, simplifying system design compared to free-space implementations.

Additionally, the interaction mechanism between light and the metasurface differs significantly. In free-space systems, the incident beam directly illuminates the metasurface nanostructures to induce modulation. In the on-chip configuration, however, the guided incident light is first selectively extracted by the metasurface, and only subsequently interacts with the nanostructures. This indirect interaction pathway necessitates careful consideration of materials that can achieve relatively high optical extraction efficiency to ensure effective modulation and overall device performance.

Specific limitations or advantages influencing design choices within the waveguide configurations: on the one hand, the on-chip metasurface platform provides notable advantages for integrated photonic systems. As the unextracted portion of the guided wave continues to propagate within the waveguide, it can be further harnessed by downstream photonic elements, enabling multi-stage cascading and the implementation

of complex on-chip optical circuits. Furthermore, the confinement of light within the waveguide suppresses the undesired zero-order diffraction, resulting in significant enhancement of the holographic image quality compared to free-space implementations. On the other hand, a notable limitation of the on-chip metasurface platform is the inherently lower extraction efficiency relative to the direct light-matter interaction in free space. Nevertheless, the achieved efficiency is sufficient for our current demonstration and proof-of-concept validation.

S6. Numerical simulation of the azimuth angle and ellipticity angle at the EP

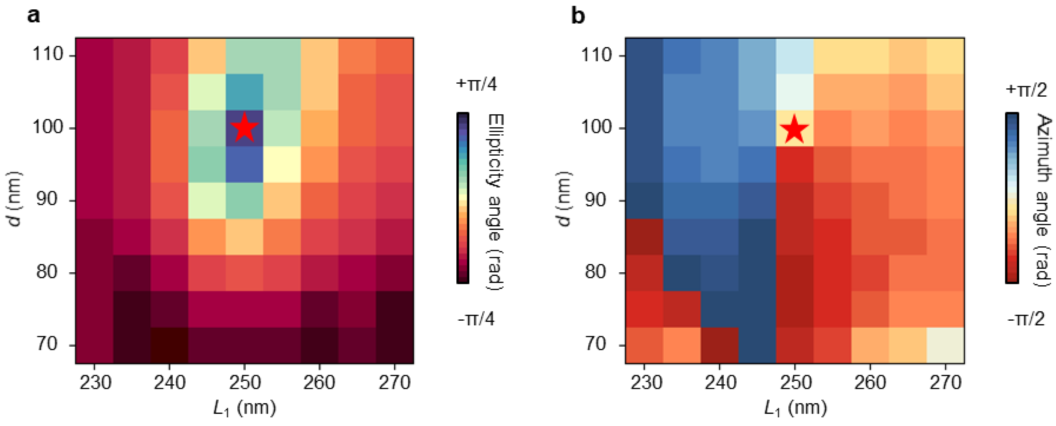

Fig. S8. Simulated ellipticity angle (**a**) and azimuth angle (**b**) of the extracted light.

Because the on-chip configuration does not permit independent incidence of LCP/RCP modes, it is not feasible to separately analyze the variation trends of the two eigenstates within the parameter space. Here, to confirm whether the proposed on-chip topological metasurface exhibits degeneracy and operates in a circularly polarized state at the EP, we analyzed the azimuth and ellipticity angles of the entire extracted light field within the parameter space (L_1, d). Slight shifts of the EP position within the parameter space are observed due to variations in the fineness of the mesh settings during simulations. However, as the focus of this study is the characteristic analysis at the EP, these minor deviations are considered acceptable. As shown in Fig. S8a and S8b, the extracted light field at the EP exhibits circular polarization, confirming that the EP undergoes degeneracy and operates in a circularly polarized state within the parameter space.

S7. Numerical simulation of PB phase of on-chip meta-atom

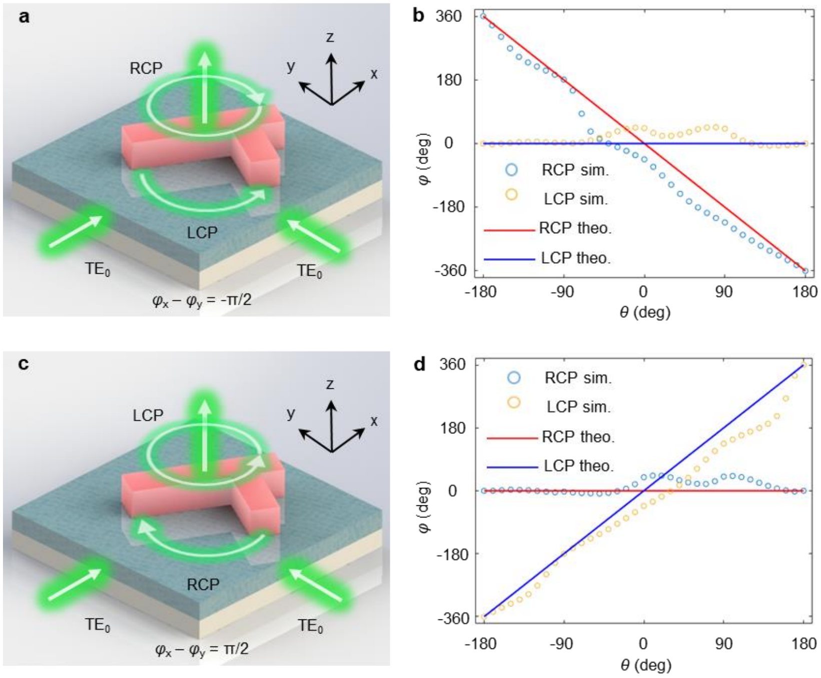

Fig. S9. a, b, Simulated phase delay for a single meta-atom integrated on the waveguide under LCP guided waves incidence. **c**, **d**, Simulated phase delay for a single meta-atom integrated on the waveguide under RCP guided waves incidence.

To clarify the PB phase, we present simulated models of the on-chip meta-atom under incident LCP and RCP guided waves, as shown in Fig. S9a and S9c, respectively. The LCP and RCP guided waves are synthesized by two orthogonally propagating TE_0 guided waves along x- and y-axes, with an initial phase offset of - $\pi/2$ or $\pi/2$. It is observed that the incident LCP/RCP waves are converted to RCP/LCP waves and undergo an additional phase shift of 2θ, while the unconverted LCP/RCP waves retain their original phase shift (Fig. S9b and S9d). Due to the insufficient resolution of the fine mesh in the simulation, some points deviate from the theoretical values.

S8. Numerical simulation results of EP in LCP and RCP components

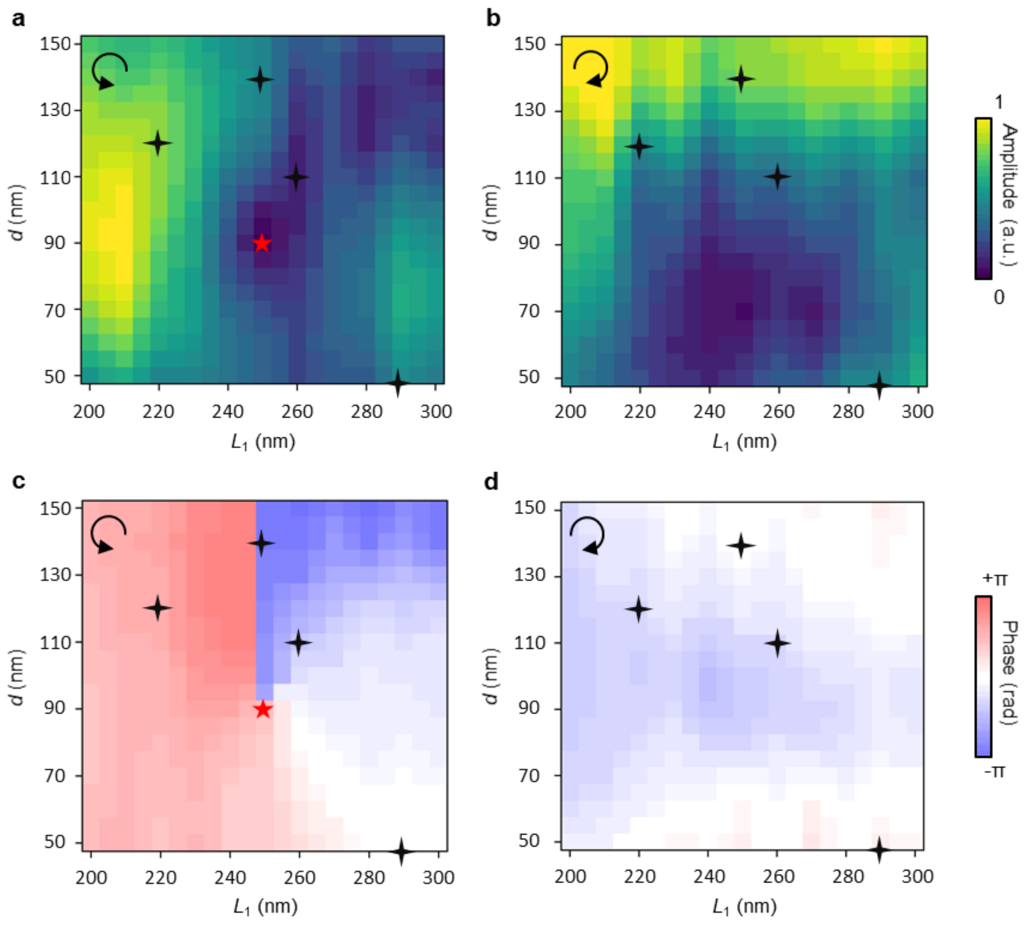

Fig. S10. a, **b**, The amplitude of the extracted LCP/RCP component from the guided waves. **c**, **d**, The phase distributions of the extracted LCP/RCP component from the guided waves.

To offer a more detailed characterization of the region near the EP, we present the simulated amplitude and phase distributions at 525 nm (see Fig. S10). For the extracted LCP component, it can be observed that at the EP (the red star), the guided waves extraction is suppressed, and we can obtain the 2π-phase accumulation by encircling the EP, known as the topological phase. The EP occurs at (250 nm, 90 nm). In contrast, for the RCP channel, no corresponding 2π-phase accumulation is observed; instead, a relatively flat phase distribution is present. The four black stars denote selected

topological meta-atoms, with corresponding geometric parameters (L_1, d) of (220 nm, 120 nm), (250 nm, 140 nm), (260 nm, 110 nm), and (290 nm, 40 nm), respectively.

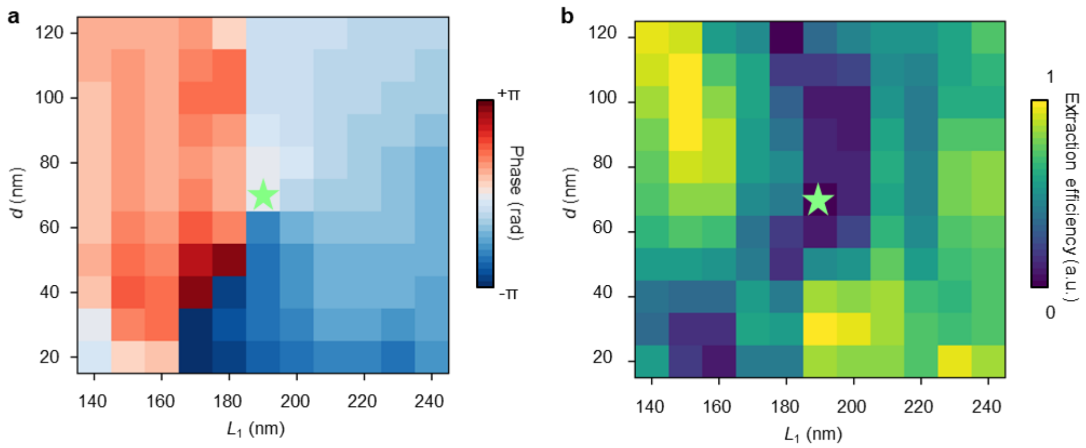

Fig. S11. Simulated topological phase (**a**) and extraction efficiency (**b**) distributions for the extracted RCP component at 505 nm.

Moreover, we have also identified an EP within the RCP channel under the same parameter space (see Fig. S11), which exhibits a topological phase behavior aligned to that observed in the LCP case, with a wavelength shift. This finding further demonstrates the robustness of our on-chip guided-wave extraction strategy for EP formation.

S9. Explanation for the Gerchberg-Saxton (GS) algorithm

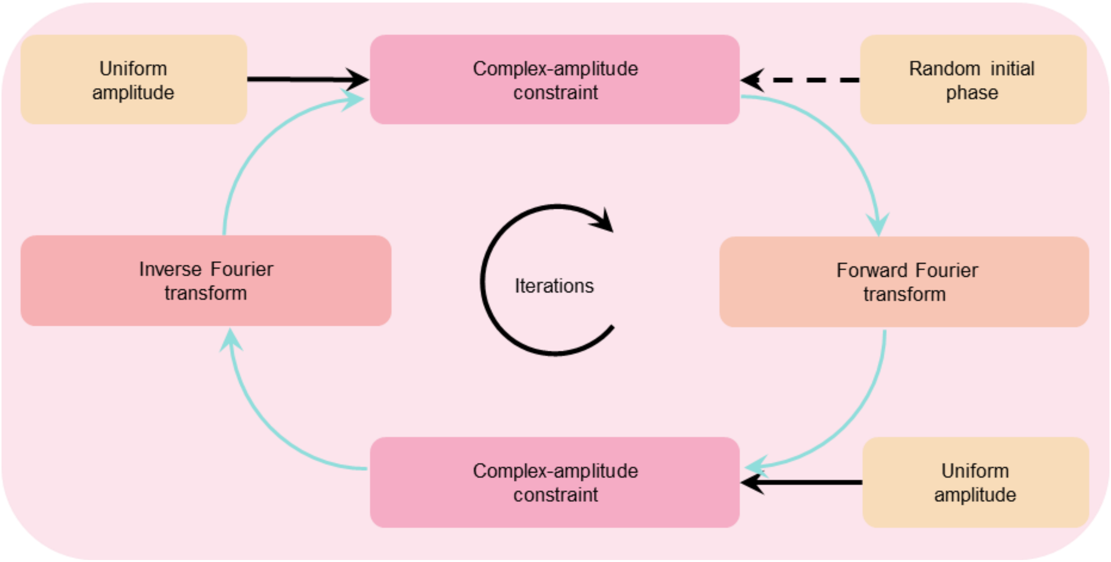

Fig. S12. Schematic flowchart of the Gerchberg-Saxton algorithm used for phase-only hologram generation.

To generate the desired phase-only meta-hologram, we employed the conventional Gerchberg-Saxton algorithm (see Fig. S12), a widely used iterative phase retrieval technique in computational holography. The algorithm iteratively enforces complex-amplitude constraints in both the hologram (Spatial) domain and the image (Fourier) domain, progressively refining the phase distribution to approximate the target intensity pattern. The procedure begins with the construction of a complex optical field in the hologram plane by combining a random initial phase with a uniform amplitude distribution, representing the incident beam. A forward Fourier transform is then applied to propagate the field to the image plane. In the Fourier domain, the amplitude is replaced with the square root of the target intensity distribution, while the phase component is preserved. The resulting complex field is then transformed back to the

hologram plane via an inverse Fourier transform. Subsequently, the amplitude is reset to the original uniform distribution while retaining the updated phase information. This iterative process is repeated for a fixed number of iterations or until a convergence criterion is reached. The final phase profile in the hologram plane is used as the phase-only hologram for the desired image reconstruction.

S10. Each step of the sample preparation process

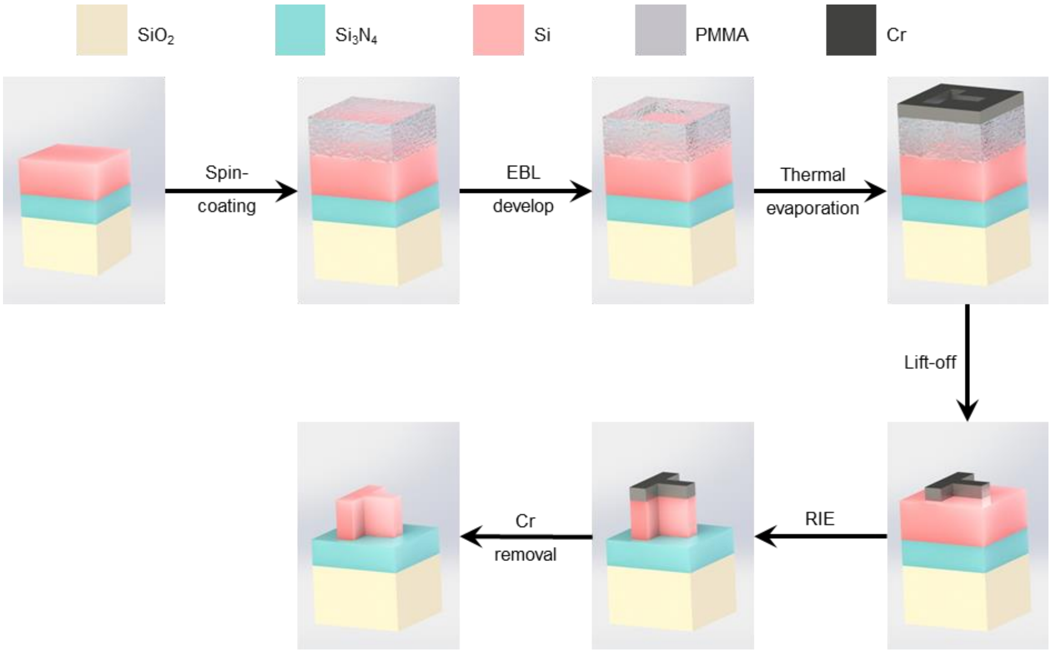

Fig. S13. Schematic illustration of the fabrication process.

The sample fabrication procedure is shown in Fig. S13 and a corresponding description of the fabrication procedure is presented in the “Methods” section of the manuscript.
